# Foundation Doctors’ Understanding of Hospital Chaplaincy as Part of Holistic Care: A Cross-Sectional Survey at a District General Hospital in the United Kingdom

**DOI:** 10.7759/cureus.116550

**Published:** 2026-09-19

**Authors:** Ho Yuen Jonathan Chiu, Geraldine Donnelly

**Affiliations:** 1 Medicine, Bolton NHS Foundation Trust, Bolton, GBR; 2 Medical Education, Bolton NHS Foundation Trust, Bolton, GBR

**Keywords:** chaplaincy, foundation doctors, holistic care, medical education, multidisciplinary care

## Abstract

Hospital chaplaincy is an important component of holistic care, but its role may not be fully understood by foundation doctors during early postgraduate training. This cross-sectional survey explored foundation doctors' awareness, perceptions, and use of hospital chaplaincy services at a district general hospital in the United Kingdom. Of 83 eligible foundation doctors, 44 completed the anonymous questionnaire (53% response rate, 95% CI: 42.4-63.4%). Although 88.6% (95% CI: 76.0-95.0%) of respondents were aware that the hospital had a chaplaincy service, 63.6% (95% CI: 48.9-76.2%) were unfamiliar with referral pathways, and half reported receiving no formal chaplaincy teaching during medical school or foundation training. These findings suggest that awareness of chaplaincy services does not necessarily translate into practical referral knowledge. As frontline clinicians, foundation doctors require an understanding of multidisciplinary support services to provide holistic patient-centered care. Thus, brief chaplaincy teaching, clearer referral guidance, and inclusion of chaplaincy within induction and multidisciplinary education may help address this educational gap.

## Introduction

Hospital chaplaincy services are part of the wider multidisciplinary support available to patients, families, and healthcare staff in National Health Service (NHS) settings in the United Kingdom (UK) [1]. While chaplaincy is commonly associated with religious support and end-of-life care, chaplains also provide broader emotional and psychosocial support within hospital practice, contributing to holistic and patient-centered care [2,3].

Foundation doctors are often among the first medical professionals encountered by patients during a hospital stay, giving them an early opportunity to identify issues that may benefit from chaplaincy support. This makes awareness of the role and referral pathways of chaplaincy services important for facilitating timely and appropriate referrals. The UK Foundation Programme curriculum recognizes the importance of holistic planning in the training of foundation doctors and includes this within the 13 professional capabilities required for Foundation Programme sign-off. Foundation Professional Capability 3 (FPC 3), under Higher-Level Outcome 1 (HLO 1), requires foundation doctors to formulate treatment plans that give ethical consideration to patients' physical, psychological, and social needs [4]. In practice, addressing these needs may require foundation doctors to have sufficient awareness of the wider multidisciplinary support available, including how and when to access services such as hospital chaplaincy.

Existing literature suggests that healthcare professionals demonstrate varying levels of understanding of hospital chaplaincy services and their broader clinical role. Notably, Ma et al. reported inconsistent awareness of chaplaincy functions among 153 staff at a UK hospital, with nearly half of the surveyed junior doctors reporting poor or no knowledge of hospital chaplaincy services [3]. Similarly, in a conference abstract, Poon et al. identified limited awareness of local chaplaincy services among both medical and oncology junior doctors within a UK district general hospital [5]. Both reports also indicated that chaplaincy services remain primarily associated with religious or end-of-life care, despite their broader contributions to holistic patient support.

Notwithstanding the increasing emphasis on multidisciplinary and patient-centered care, studies that have specifically examined foundation doctors’ awareness and understanding of hospital chaplaincy services during early postgraduate training appear lacking. Given the frontline role of foundation doctors and the objectives of the Foundation Programme in the UK context, this study aimed to assess foundation doctors' prior educational exposure to chaplaincy, awareness of chaplaincy services and referral pathways, previous use of chaplaincy in clinical practice, and perceptions and confidence regarding its role through a cross-sectional survey, and to identify potential implications for chaplaincy education and multidisciplinary practice.

## Materials and methods

Study design and participants

A cross-sectional survey of Foundation Year 1 (FY1) and Foundation Year 2 (FY2) doctors was conducted at the Royal Bolton Hospital (Bolton NHS Foundation Trust), Farnworth, Bolton, UK, over a four-week period in May 2026. All 83 FY1 and FY2 doctors employed at the trust during this period, as identified from the Foundation Programme administrator's records, were eligible to participate, with no exclusions applied for rotation status or leave. An anonymous questionnaire was distributed electronically via Microsoft Forms (Microsoft Corporation, Redmond, Washington, United States, supplemented by promotion through foundation doctor teaching sessions and the foundation doctors' WhatsApp group (Meta Platforms, Inc., Menlo Park, California, United States). As all eligible foundation doctors were invited to participate, recruitment represented an attempted census of the local foundation doctor population; however, voluntary participation introduced the potential for self-selection and non-response bias. One reminder was sent midway through the survey period via the foundation doctors' WhatsApp group. Participation was voluntary, and no identifiable personal information was collected.

Survey instrument

The questionnaire was developed by the primary researcher, with previously published studies of junior doctors' awareness of hospital chaplaincy services [3,5] used as background rather than as sources of adapted items. All items were investigator-generated. Prior to distribution, the questionnaire was reviewed by the head chaplain and supervising consultant for face validity. The first three FY1 respondents then completed the questionnaire as part of the ongoing distribution, after which their feedback on item clarity was sought. As no changes to item wording were made following this feedback, their responses were subsequently retained within the final analytic sample (n=44).

The questionnaire consisted of 14 items assessing four distinct domains: (i) participants’ current training grade and prior educational exposure to chaplaincy; (ii) awareness of local chaplaincy services and referral pathways; (iii) previous utilization of chaplaincy services in clinical practice; and (iv) perceptions of chaplaincy within holistic and multidisciplinary care. Items consisted of binary, multiple-choice, ordinal, and five-point Likert-scale formats. The five-point Likert-type items were analyzed individually rather than combined into a composite score, as they assessed distinct aspects of perceptions of chaplaincy. In one question, participants could select one or more options that applied; responses to this item were analyzed as the percentage of respondents selecting each option, with a denominator of 44, distinct from the single-response items. One of the 14 items was an optional free-text question allowing respondents to provide additional comments regarding chaplaincy awareness and educational support.

Data analysis

Survey responses were analyzed in Microsoft Excel (Microsoft Corporation) using descriptive statistics, including frequencies and percentages. 95% confidence intervals for selected key proportions were calculated using the Wilson score method to indicate the precision of these estimates. As the study was exploratory and descriptive, with no prespecified hypothesis or comparative primary outcome, no hypothesis tests or inferential between-group comparisons were performed. The five-point Likert-type items were analyzed individually and were not combined into a composite score.

Optional free-text responses were analyzed using an inductive descriptive content analysis approach. All responses were reviewed by the primary researcher, who identified recurring ideas and grouped similar responses into descriptive categories. These categories were subsequently consolidated into recurring themes. Responses could contribute to more than one descriptive category, and some responses did not clearly align with a specific theme. Accordingly, theme counts were not mutually exclusive and did not necessarily sum to the total number of free-text respondents. Coding was performed by the primary researcher and was not independently double-coded. The free-text component was intended to supplement the quantitative findings rather than constitute a standalone qualitative analysis.

A post hoc exploratory descriptive comparison of selected outcomes by training grade (FY1 versus FY2) was also undertaken using frequencies and percentages. Confidence intervals were not calculated for these subgroup comparisons, which were intended as descriptive, exploratory analyses only; no hypothesis testing was performed.

Ethical considerations

The study was reviewed by the Clinical Audit and Effectiveness, Bolton NHS Foundation Trust, which concluded that, as the study was conducted as a staff questionnaire survey within the hospital, this was not considered as ‘research’ and did not require formal ethical approval. 

## Results

Participants’ academic information and prior educational exposure to chaplaincy

A total of 44 responses were received from 83 eligible foundation doctors, yielding a 53% response rate (95% CI: 42.4-63.4%). Respondents comprised 26 FY1 doctors (59.1%) and 18 FY2 doctors (40.9%).

Of those surveyed, educational exposure to chaplaincy was notably limited. Half of the respondents (22/44, 50%, 95% CI: 35.8-64.2%) reported receiving no formal teaching on hospital chaplaincy during either medical school or foundation training. Ten respondents (22.7%) reported receiving formal teaching during foundation training only, five (11.4%) during medical school only, two (4.5%) during both medical school and foundation training, and five (11.4%) were unsure.

Awareness of local chaplaincy services and referral pathways

The majority of respondents (n=39, 88.6%, 95% CI: 76.0-95.0%) were aware that the hospital had a chaplaincy service. However, a total of 28 doctors (63.6%, 95% CI: 48.9-76.2%) did not know (n=19, 43.2%) or were not sure (n=9, 20.5%) how to contact chaplaincy or the referral pathways, compared to 16 respondents (36.4%) who reported knowing how to do so.

Previous utilization of chaplaincy services in clinical practice

Regarding clinical use, only 13 respondents (29.5%, 95% CI: 18.2-44.2%) reported having ever referred a patient to chaplaincy during foundation training.

Perceptions of chaplaincy within holistic and multidisciplinary care

Confidence in recognizing situations where chaplaincy support may be appropriate was mixed. Sixteen respondents (36.4%) reported being either unconfident or very unconfident, while 13 (29.5%) reported being confident or very confident. A further 15 respondents (34.1%) selected a neutral response.

Understanding of the broader role of chaplaincy beyond end-of-life care was also variable. Only seven respondents (15.9%) reported that they understood the broader role of hospital chaplaincy, while 14 (31.8%) reported partial understanding. In contrast, 16 respondents (36.4%) reported no understanding, and seven (15.9%) were unsure.

**Figure 1:**
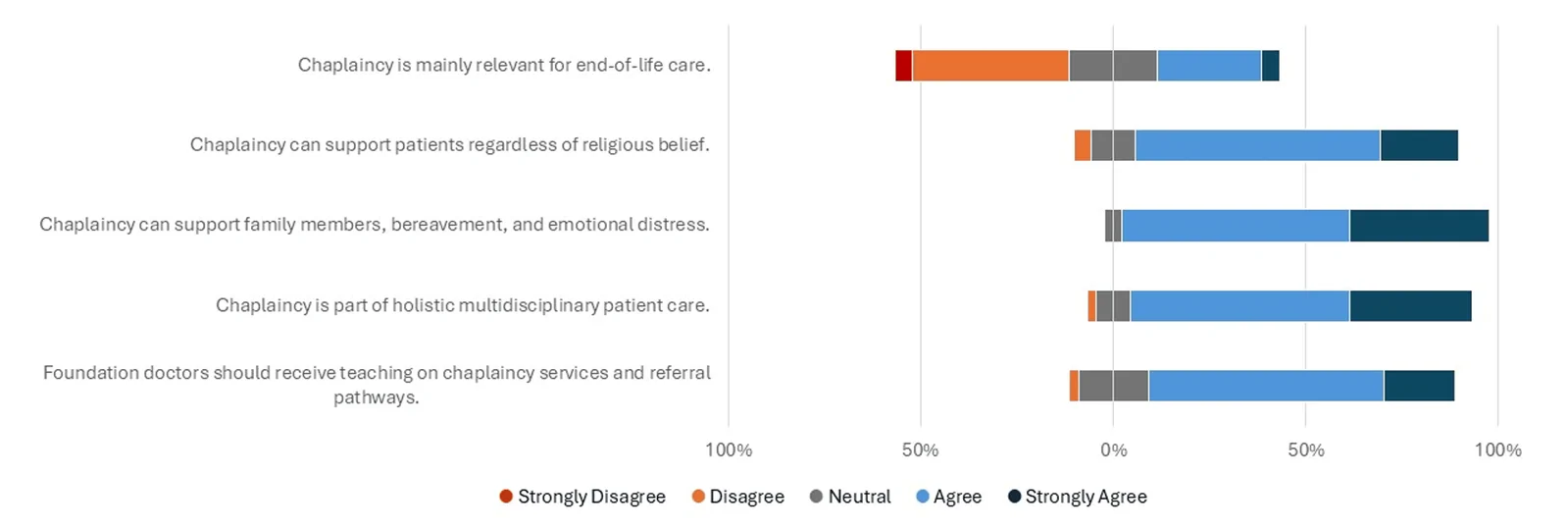
Foundation doctors’ perceptions of hospital chaplaincy roles (N=44) Responses are shown across five Likert categories: strongly disagree, disagree, neutral, agree, and strongly agree.

When specific chaplaincy roles were presented, respondents generally recognized the wider value of chaplaincy. Most respondents agreed or strongly agreed that chaplaincy can support patients regardless of religious belief (n=37, 84.1%), support family members, provide bereavement and emotional support (n=42, 95.5%), and contribute to holistic multidisciplinary patient care (n=39, 88.6%). In addition, 35 respondents (79.5%, 95% CI: 65.5-88.8%) agreed or strongly agreed that foundation doctors should receive teaching on chaplaincy services and referral pathways. In particular, responses to the statement that chaplaincy is mainly relevant for end-of-life care were more mixed (n=14, 31.8% agreeing; n=20, 45.5% disagreeing; n=10, 22.7% neutral). Full distributions across all five Likert response categories (strongly disagree, disagree, neutral, agree, and strongly agree) for each statement are presented in Figure 1.

When asked to identify situations in which chaplaincy support may be appropriate, participants could select all applicable options. As summarized in Table 1, the most commonly selected indications were end-of-life care (n=42, 95.5%), religious or spiritual requests (n=39, 88.6%), and bereavement support (n=37, 84.1%). Broader indications were selected less frequently, including emotional distress (n=24, 54.5%), family distress (n=21, 47.7%), ethical or existential distress (n=19, 43.2%), and staff support (n=15, 34.1%).

**Table 1:** Situations for chaplaincy involvement (N=44) NOTE: multiple responses were permitted

Situation	Frequency (Percentage)
End-of-life care	42 (95.5%)
Religious/spiritual request	39 (88.6%)
Bereavement support	37 (84.1%)
Emotional distress	24 (54.5%)
Family distress	21 (47.7%)
Ethical or existential distress	19 (43.2%)
Staff support	15 (34.1%)
Would not usually consider chaplaincy	1 (2.3%)

Open-ended responses were consistent with the quantitative findings. Among the 44 doctors surveyed, 26 (59.1%) elaborated their thoughts in the comment field. Analysis identified two recurring themes, with an additional suggestion relating to service visibility.

Theme 1

Need for formal teaching on chaplaincy roles and referral processes. This was the most commonly identified theme, raised by 18 of the 26 respondents, and centered on the need for formal teaching on the role of chaplaincy and when and how to refer. For example:

'*Teaching on chaplaincy service and the role of the chaplaincy*' (Respondent 6)

'*Teaching on when to refer and how to*' (Respondent 2)

'*Maybe teaching on referral processes*' (Respondent 36)

Theme 2

Need for clarity on contact pathways and scope of support. Raised by five respondents, comments reflected uncertainty about what support chaplaincy provides and who can access it, for example:

'*Signposting contact information*' (Respondent 22)

'*How to get in touch and the scope of support they can offer*' (Respondent 39)

One respondent specifically raised questions about scope and religious coverage:

'*What they can offer? Who for? How to contact? Which religions?*' (Respondent 41)

Additional suggestion: Improving visibility of the service. One respondent suggested using a physical prompt to increase awareness of when chaplaincy could be accessed:

'*Maybe a poster on circumstances where they can be called apart from end of life*' (Respondent 11)

Comparison by training grade

As a secondary exploratory analysis, selected outcomes were compared descriptively between FY1 (n=26) and FY2 (n=18) respondents. Awareness that the hospital had a chaplaincy service was high in both groups (FY1: 24/26, 92.3%; FY2: 15/18, 83.3%). A higher proportion of FY2 respondents reported knowing how to contact or refer to chaplaincy (8/18, 44.4%) compared with FY1 respondents (8/26, 30.8%), and previous referral to chaplaincy during foundation training was also more common among FY2 respondents (6/18, 33.3%) than FY1 respondents (7/26, 26.9%). FY2 respondents were also less likely to report having received no formal chaplaincy teaching (6/18, 33.3%) than FY1 respondents (16/26, 61.5%).

In contrast, self-reported confidence in recognizing situations where chaplaincy support may be appropriate was lower among FY2 respondents (4/18, 22.2% confident or very confident) than FY1 respondents (9/26, 34.6%). Understanding of the broader role of chaplaincy beyond end-of-life care was similar between the two groups (FY1: 4/26, 15.4%; FY2: 3/18, 16.7%).

These subgroup comparisons were exploratory and descriptive only; no inferential statistical testing was undertaken given the small subgroup sizes.

## Discussion

Traditional conceptualization of hospital chaplaincy

Foundation doctors in this study are largely aware of the existence of hospital chaplaincy services, but their understanding appeared anchored to more traditional contexts. The most commonly identified situations for chaplaincy involvement were end-of-life care, religious or spiritual requests, and bereavement support, while broader indications including emotional distress, family distress, ethical or existential distress, and staff support, were selected less frequently. This matches prior findings that healthcare professionals often understand chaplaincy narrowly, mainly through religious or end-of-life care [3,5,6].

Working on the frontline, foundation doctors are at the interface between patients, families, nursing staff, senior clinicians, and wider hospital services. They are regularly involved in acute admissions, ward-based deterioration, family communication, discharge planning, and end-of-life discussions. During this routine clinical work, they can be the first clinicians to recognize patients’ unmet emotional, social needs, or family concerns. Their understanding of chaplaincy, therefore, carries direct practical relevance: foundation doctors who only see chaplaincy as a service for religious requests or end-of-life care may be less likely to consider referrals for patients experiencing emotional distress, family-related concerns, or for staff requiring well-being support.

Chaplaincy referral pathways and practical service knowledge

A noticeable discrepancy was identified between general awareness of chaplaincy services and practical knowledge of how to access them. While 88.6% of respondents were aware of the chaplaincy service in the hospital, 63.6% were unfamiliar or uncertain about how to contact or refer to it, and fewer than one third had previously made a referral to chaplaincy during foundation training. This points to a specific impediment: doctors know the service exists but do not know how to use it. Similar practical barriers have been reported more broadly in UK junior doctors, including uncertainty regarding which specialty to contact, how to contact it, and what information to include when making interspecialty referrals [7].

Awareness that a service exists does not necessarily translate into knowledge of when or how to utilize it in practice. For foundation doctors working in acute hospital environments, referral decisions may be influenced by practical familiarity, perceived relevance, and ease of access. If chaplaincy is not visible within clinical pathways, handovers, induction teaching, or ward-based multidisciplinary discussions, it may remain peripheral despite being available. As a result, opportunities for timely supportive input may be missed, particularly in situations outside obvious end-of-life or religious contexts.

The findings may provide insights into how foundation doctors learn to navigate multidisciplinary support services within hospital practice. Foundation doctors not only acquire clinical knowledge and procedural competence, but also develop an understanding of hospital workflows, including referral pathways, escalation processes, multidisciplinary roles, and available support services. This matches broader evidence that postgraduate doctors learn much of what they know through workplace experience [8]. In this context, chaplaincy awareness can be understood not as an isolated knowledge item, but as an integral part of the broader practical knowledge of hospital systems relevant to delivering holistic and patient-centered care. Early exposure to chaplaincy services may further cultivate an understanding of multidisciplinary support structures that could be transferable across different clinical settings.

The exploratory comparison by training grade revealed a mixed pattern. Higher proportions of FY2 than FY1 respondents reported knowing how to contact or refer to chaplaincy, having previously made a referral, and having received formal teaching. Greater familiarity with referral pathways and previous referral experience among FY2 respondents may be consistent with greater clinical exposure; however, this was not accompanied by higher confidence or greater understanding of chaplaincy beyond end-of-life care. Overall, FY2 respondents did not demonstrate consistently greater self-reported understanding of chaplaincy than FY1 respondents. Given the small subgroup sizes, these descriptive differences should be interpreted cautiously and may reflect sampling variation rather than a meaningful effect of training grade.

Referral knowledge and service visibility are modifiable barriers, and relatively straightforward educational interventions could address them. Targeted strategies include brief foundation teaching, inclusion of more explicit information about chaplaincy services in induction materials, clear intranet or ward-based referral information, and explicit signposting during relevant clinical teaching on end-of-life care, communication, bereavement, or holistic assessment. Education should not simply inform foundation doctors that chaplaincy exists but should translate awareness into usable clinical knowledge: when chaplaincy may be helpful, how to contact the service, what support it can provide, and how it fits within wider multidisciplinary care. This may help embed chaplaincy more effectively within foundation doctors’ clinical practice.

Educational implications for foundation training

The data revealed limited formal educational exposure to chaplaincy services, with half of respondents reporting no teaching on the subject during either medical school or foundation training. This may partly explain the inconsistent understanding of chaplaincy observed among respondents. In the absence of structured teaching, foundation doctors' understanding may develop unevenly through clinical exposure. This echoes findings that junior doctors often engage inconsistently with patients' spiritual and religious needs, sometimes feeling unprepared to do so [6]. If such exposure occurs predominantly during end-of-life care, religious requests, or bereavement situations, it may reinforce a narrower perception of chaplaincy rather than its broader contribution to holistic care. As a result, appropriate referrals and the potential benefits of a broader understanding of chaplaincy services, including staff support, debriefing following difficult clinical events, and broader pastoral, spiritual and religious care may be limited [1].

The widespread endorsement of formal chaplaincy and referral training among respondents supports a perceived need for this educational content. Importantly, a pragmatic educational approach need not require substantial curriculum redesign; a brief, localized session during foundation induction or protected teaching could clarify the scope of chaplaincy, exemplify appropriate referral situations, and enhance understanding of its integration in multidisciplinary care [3,6]. These concepts could be incorporated into established curricula on communication, palliative care, bereavement, professionalism, or holistic assessment, fostering a more comprehensive understanding.

Given that holistic planning is a core element of the Foundation Programme, future research employing a pre- and post-intervention design could evaluate a brief chaplaincy orientation delivered during foundation teaching, using a format similar to short departmental orientation visits such as pathology laboratory tours, which combine service familiarization with practical guidance on when and how to access the service. This would include a short overview of chaplaincy's role and scope, examples of appropriate referral situations, clear guidance on how to contact the service, a brief introduction to chaplaincy facilities, and an opportunity for questions. The intervention could be evaluated using pre- and post-session measures of awareness, referral-pathway knowledge, confidence in identifying appropriate referral situations, and understanding of the broader role of chaplaincy, with subsequent referral activity assessed after a defined follow-up period. Such an approach would move beyond descriptive awareness data and help determine whether a focused educational intervention improves foundation doctors' knowledge, confidence, and practical engagement with chaplaincy services.

Strengths and limitations

This study addresses a focused and relatively underexplored area of early postgraduate medical practice: foundation doctors’ awareness and understanding of hospital chaplaincy services within holistic care. Strengths include participation from 53% of eligible foundation doctors, although, as noted below, this may partly reflect selective engagement from more interested respondents given the recruitment approach. Inclusion of both FY1 and FY2 doctors provided insights spanning the entire spectrum of foundation training. The questionnaire examined multiple relevant domains, including educational exposure, referral knowledge, previous use, confidence, and perceptions of chaplaincy roles. The addition of the optional free-text question also provided contextual insights into respondents’ perceived educational needs.

Limitations include the single-center design and modest sample size (n=44), which may limit generalization to other institutions, training environments, or other multidisciplinary support services beyond chaplaincy. Due to the voluntary nature of participation, self-selection response bias cannot be ruled out since doctors with strong views towards chaplaincy may be more inclined to respond. Responses may also have been influenced by social desirability bias, particularly for items concerning the perceived value of chaplaincy and further teaching. Moreover, the questionnaire relied on self-reported measures of awareness, confidence, and prior use rather than objective assessment of knowledge or observed referral behavior. Additionally, despite preliminary review by the head chaplain and clinical expert, the questionnaire was developed for local use and lacked formal validation, which may limit the overall reliability of the findings. Finally, the cross-sectional design can identify awareness gaps and educational needs but cannot establish causality between teaching exposure and confidence or understanding. It is also possible that doctors with greater confidence were more likely to have sought out or recalled prior teaching.

## Conclusions

This study highlights a practical gap in foundation doctors’ engagement with hospital chaplaincy services. While chaplaincy was generally recognized as an available service, its role appeared to be understood mainly through traditional end-of-life, religious, and bereavement contexts. Broader supportive functions were less consistently recognized, and many respondents lacked confidence or practical knowledge regarding referral.

Bridging this gap may require a multifaceted approach: brief foundation teaching, clearer referral signposting, and inclusion of chaplaincy within induction or multidisciplinary education. However, this study did not evaluate the effectiveness of these interventions. Future work using a pre- and post- intervention design could test whether targeted education or improved signposting improves referral knowledge, confidence, and engagement with chaplaincy services in routine clinical practice.

## Appendices

Study questionnaire

1) What is your current grade?

Options: FY1, FY2

2) Have you received formal teaching on hospital chaplaincy services during medical school or foundation training?

Options: Medical school only, Foundation training only, Both, Neither, Unsure

3) Before this survey, were you aware that the hospital has a chaplaincy service?

Options: Yes, No

4) Do you know how to contact/refer to chaplaincy?

Options: Yes, No, Unsure

5) Have you ever involved or referred patients to the hospital chaplaincy service for patient care, family support, or staff support during foundation training?

Options: Yes, No

6) How confident are you in recognizing situations where chaplaincy support may be appropriate?

Options: Very confident, Confident, Neutral, Unconfident, Very unconfident

7) Do you feel you understand the role of hospital chaplaincy services beyond end-of-life care?

Options: Yes, Partly, No, Unsure

8) Chaplaincy is mainly relevant for end-of-life care.

Options: Strongly disagree, Disagree, Neutral, Agree, Strongly agree

9) Chaplaincy can support patients regardless of religious belief.

Options: Strongly disagree, Disagree, Neutral, Agree, Strongly agree

10) Chaplaincy can support family members, bereavement, and emotional distress.

Options: Strongly disagree, Disagree, Neutral, Agree, Strongly agree

11) Chaplaincy is part of holistic multidisciplinary patient care.

Options: Strongly disagree, Disagree, Neutral, Agree, Strongly agree

12) Foundation doctors should receive teaching on chaplaincy services and referral pathways.

Options: Strongly disagree, Disagree, Neutral, Agree, Strongly agree

13) In which situations would you most likely consider involving chaplaincy? (Select all that apply)

Options: End-of-life care, Bereavement support, Religious/spiritual request, Emotional distress, Family distress, Staff support, Ethical or existential distress, I would not usually consider chaplaincy

14) Any comments on how foundation doctors could be better supported to understand or access chaplaincy services? (Open-ended question)
